# End-to-End Architecture for Real-Time IoT Analytics and Predictive Maintenance Using Stream Processing and ML Pipelines

**DOI:** 10.3390/s25092945

**Published:** 2025-05-07

**Authors:** Ouiam Khattach, Omar Moussaoui, Mohammed Hassine

**Affiliations:** 1 Department of Informatics, MATSI Laboratory EST, University Mohammed First, Oujda 60000, Morocco; o.moussaoui@ump.ac.ma; 2 Tisalabs Limited, T12 Y275 Cork, Ireland; mo.hassine@tisalabs.com

**Keywords:** predictive maintenance, internet of things, real-time data, machine learning pipeline

## Abstract

The rapid proliferation of Internet of Things (IoT) devices across industries has created a need for robust, scalable, and real-time data processing architectures capable of supporting intelligent analytics and predictive maintenance. This paper presents a novel comprehensive architecture that enables end-to-end processing of IoT data streams, from acquisition to actionable insights. The system integrates Kafka-based message brokering for the high-throughput ingestion of real-time sensor data, with Apache Spark facilitating batch and stream extraction, transformation, and loading (ETL) processes. A modular machine-learning pipeline handles automated data preprocessing, training, and evaluation across various models. The architecture incorporates continuous monitoring and optimization components to track system performance and model accuracy, feeding insights to users via a dedicated Application Programming Interface (API). The design ensures scalability, flexibility, and real-time responsiveness, making it well suited for industrial IoT applications requiring continuous monitoring and intelligent decision-making.

## 1. Introduction

Predictive maintenance (PDM) has emerged as a vital application within the IoT ecosystem, enabling the early detection of equipment anomalies and minimizing unplanned downtime [1]. By analyzing data from sensors embedded in physical assets, PDM enhances system reliability, reduces maintenance costs, and improves overall operational efficiency [2]. Unlike traditional strategies such as reactive maintenance (performed after failure) or preventive maintenance (scheduled regardless of equipment condition), PDM leverages real-time data to monitor equipment health and schedule interventions only when necessary. This condition-based approach not only extends the lifespan of machinery but also ensures more efficient resource utilization.

However, deploying PDM in real-world IoT environments presents notable challenges. IoT systems produce continuous, high-frequency data from distributed sources, requiring robust architectures for real-time data ingestion, transformation, storage, and analysis. Scalable data processing technologies and AI-driven models must be seamlessly integrated to support continuous learning and feedback loops.

This paper proposes a modular and scalable end-to-end architecture tailored for real-time predictive maintenance in IoT settings. The design incorporates modern tools for data processing, machine learning (ML) lifecycle management, and performance monitoring, ensuring a reliable and adaptive system capable of handling the demands of industrial-scale deployments.

- Apache Kafka is used for high-throughput, fault-tolerant data ingestion from multiple IoT sensors and producers.
- Apache Spark handles batch and stream processing to perform ETL on incoming data.
- InfluxDB, a time-series database, stores sensor data for efficient retrieval and historical trend analysis.
- A flexible machine learning pipeline, managed by MLflow, automates data preprocessing, model training, and experiment tracking. The pipeline supports a range of models.
- Monitoring and optimization modules continuously track model performance and system metrics, ensuring that predictions remain accurate and the system operates efficiently.

The proposed architecture is adaptable to various industrial domains, including manufacturing, energy [3], transportation, and smart infrastructure [4]. It supports real-time analytics and continuous model improvement, making it an ideal foundation for intelligent predictive maintenance solutions in complex IoT ecosystems.

The rest of this paper is organized as follows: Section 2 reviews related work and existing solutions; Section 3 describes the proposed architecture in detail; Section 4 discusses implementation considerations and benefits; Section 5 concludes with future directions.

## 2. Related Works

This section provides an overview of the foundational technologies underpinning our proposed architecture. Section 2.1 covers Apache Kafka. Section 2.2 explores Apache Spark’s capabilities for real-time data processing. Section 2.3 discusses machine learning algorithms optimized for predictive maintenance applications.

### 2.1. Apache Kafka

Apache Kafka [5] is a distributed event streaming platform designed to manage real-time data pipelines and large-scale streaming applications. Known for its high throughput and low latency, Kafka is widely used in domains such as log aggregation, microservices communication [6], and real-time analytics [7]. Its architecture makes it particularly well suited for environments that require the reliable processing of high-frequency data streams, such as predictive maintenance in IoT systems.

**Figure 1:**
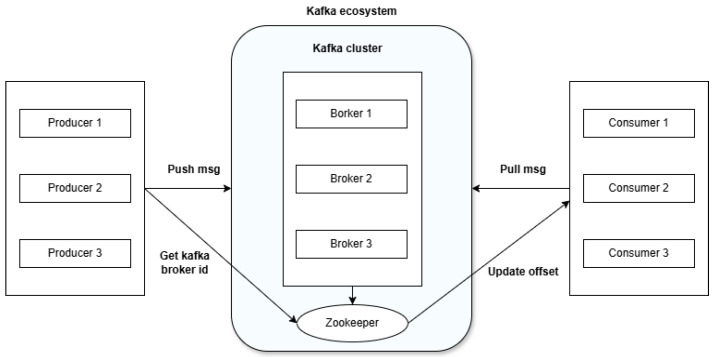
Apache Kafka architecture.

Kafka operates on a distributed publish–subscribe model as demonstrated in Figure 1. Producers send messages to topics, which are divided into partitions to enable parallel processing. These partitions are distributed across multiple brokers within a Kafka cluster, ensuring scalability and fault tolerance. Consumers pull messages from these topics, and consumer groups allow for balanced processing across multiple instances. Kafka ensures high availability through data replication; each partition has a leader and one or more replicas across different brokers.

Integrating Kafka into the system offers several important benefits. First, its real-time streaming capabilities allow for low-latency data transmission, which is essential for the early detection of anomalies and timely failure prediction [8]. Second, Kafka’s distributed design supports horizontal scalability, enabling the architecture to accommodate increasing volumes of sensor data without performance degradation [9]. Third, its fault-tolerant architecture ensures data durability and availability, even in the event of hardware or network failures.

Moreover, Kafka helps decouple system components by separating data producers from consumers, enhancing modularity and maintainability. This decoupling allows ETL processes, analytics modules, and machine learning pipelines to evolve independently. Kafka’s replay capability further supports historical data reprocessing, which is valuable for model retraining, audits, and failure diagnosis. Finally, Kafka integrates seamlessly with stream processing frameworks such as Apache Flink and Kafka Streams, facilitating real-time feature extraction, enrichment, and inference within the data pipeline.

Collectively, these features make Apache Kafka a powerful and essential component of the proposed architecture, supporting a scalable, reliable, and intelligent predictive maintenance solution for industrial IoT environments.

### 2.2. Apache Spark for Real-Time Data Preprocessing

Apache Spark [10] is an open-source, distributed computing system that has become the industry standard for large-scale data processing. Developed at the University of California, Berkeley, Spark significantly outperforms earlier big data frameworks through its in-memory computation model and optimized execution engine.

Spark excels in real-time data processing through its Structured Streaming component, which provides a scalable, high-throughput, fault-tolerant stream processing framework. This component implements a micro-batch processing model that divides incoming data streams into small, manageable batches for near-instantaneous processing. The framework’s seamless integration with Kafka makes it ideal for consuming high-velocity IoT sensor data, including system metrics such as CPU usage, memory consumption, temperature, and power levels.

When processing real-time data streams, the freshness of information becomes a critical factor, especially for time-sensitive applications like predictive maintenance. The Age of Information (AoI) metric plays a vital role by quantifying data freshness as the time elapsed since the data’s generation at its source [11]. Spark’s Structured Streaming is designed to minimize AoI through its low-latency processing capabilities, ensuring that analytics and decisions are based on the most current data available. This is particularly important for IoT sensor networks, where stale data can lead to missed failure indicators or unnecessary maintenance interventions.

The Spark ecosystem encompasses several specialized libraries that significantly extend its functionality [12]. Spark Streaming delivers scalable, fault-tolerant processing of continuous data streams, enabling real-time analytics and event detection. The robust MLlib library implements distributed versions of popular machine learning algorithms, offering tools for classification, regression, clustering, and anomaly detection that scale to massive datasets. Spark SQL provides a powerful interface for structured data processing through standard SQL queries, while maintaining Spark’s performance advantages. Finally, GraphX enables complex network analysis through its distributed graph computation engine, supporting algorithms for pathfinding, community detection, and influence analysis—capabilities crucial for understanding complex system dependencies.

For our predictive maintenance architecture, Spark’s unified programming model across batch and streaming workloads provides exceptional flexibility, allowing the same codebase to handle both historical data analysis and real-time processing. This unification simplifies development and maintenance while ensuring consistent data processing across different time horizons.

### 2.3. Machine Learning Algorithms for Predictive Maintenance

Machine learning algorithms have transformed predictive maintenance capabilities by enabling the early identification of potential equipment failures and the optimization of maintenance schedules [13,14]. These algorithms analyze patterns in sensor data to predict failures before they occur, minimizing downtime and reducing maintenance costs.

The Support Vector Machine (SVM) [15], a prominent supervised learning algorithm firmly grounded in statistical learning theory, consistently demonstrates superior classification performance compared to alternative methods [16]. SVMs excel in scenarios with well-defined decision boundaries between normal operation and pre-failure states, particularly when detecting anomalies that represent clear deviations from normal operating conditions.

Long Short-Term Memory (LSTM) networks [17] have shown exceptional performance in predictive maintenance due to their ability to model complex temporal dependencies in sensor data. By retaining information over extended time periods, LSTMs can detect subtle degradation patterns that might escape traditional algorithms. Bidirectional LSTMs (bi-LSTMs) [18] enhance standard LSTM performance by processing time-series data in both forward and backward directions, capturing a more comprehensive temporal context. This bidirectional approach improves the detection of complex failure signatures that depend on both past and future contexts.

Self-Supervised Learning (SSL) [19] has emerged as a promising approach for predictive maintenance scenarios with limited labeled failure data. SSL utilizes pretext tasks to derive supervision signals from unlabeled data, enabling models to learn rich representations without manual annotation. While SSL has achieved significant success in Computer Vision (CV) and Natural Language Processing (NLP) [20], its application to time-series data requires domain-specific adaptations [21,22]. Recent research has demonstrated SSL’s effectiveness in learning transferable representations from sensor data that can be fine-tuned for specific failure prediction tasks using minimal labeled examples.

Ensemble techniques combine predictions from multiple base learners to improve overall performance. Approaches such as Gradient Boosting Machines (GBMs) and eXtreme Gradient Boosting (XGBoost) have demonstrated high accuracy and robustness in predictive maintenance applications [23], particularly when dealing with imbalanced datasets where failure instances are rare compared to normal operation data. These methods are particularly advantageous in scenarios with imbalanced datasets, a common challenge in predictive maintenance applications.

Hybrid machine learning models, specifically combinations like CNN-LSTM and LSTM-SVM, have been effectively deployed for predictive maintenance functions to enhance cost efficiency and machine availability in manufacturing plants [24,25,26]. These hybrid approaches typically use one algorithm for feature extraction and another for classification or regression, resulting in more accurate failure predictions across diverse equipment types and failure modes.

The selection of appropriate algorithms depends on specific application requirements, including data characteristics, failure types, prediction horizons, and available computational resources. Our proposed architecture incorporates a flexible machine learning pipeline that supports experimentation with multiple algorithms to identify optimal approaches for different industrial contexts.

## 3. Methodology and System Architecture

Our proposed system is designed to handle the entire data lifecycle, from data extraction and preprocessing to analysis and real-time decision-making. This section describes the end-to-end architecture of the system, highlighting the ETL, machine learning pipeline in real-time predictive maintenance, and monitoring and optimization.

**Figure 2:**
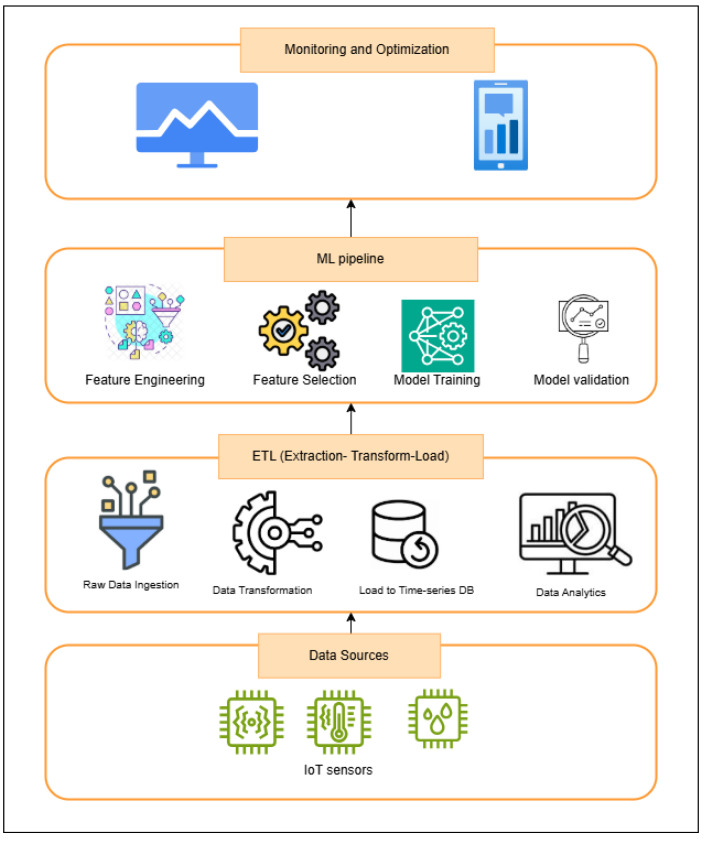
Overview of the real-time predictive maintenance pipeline for IoT systems.

The proposed system for IoT prediction in real-time consists of four layers, as depicted in Figure 2: (1) IoT data acquisition, (2) ETL processing, (3) machine learning pipeline (including feature engineering, model training, and validation), and (4) a monitoring interface for deployment feedback and optimization.

The proposed architecture for real-time predictive maintenance integrates IoT sensors with a Kafka-driven data ingestion pipeline, Spark Streaming for ETL processing, and InfluxDB as a time-series database for structured data storage. IoT devices send unstructured data to Kafka, which is consumed in real-time and processed into batches by Spark before being stored in InfluxDB.

The ML pipeline leverages the stored data for Exploratory Data Analysis (EDA), preprocessing, feature engineering, and the training of machine learning models using frameworks like MLflow, incorporating algorithms. Monitoring and optimization are supported via metrics dashboards using Grafana and Prometheus, ensuring system health and reliability. This architecture emphasizes scalability, real-time processing, and adaptability for predictive maintenance applications.

### 3.1. Real-Time ETL Pipeline

**Figure 3:**
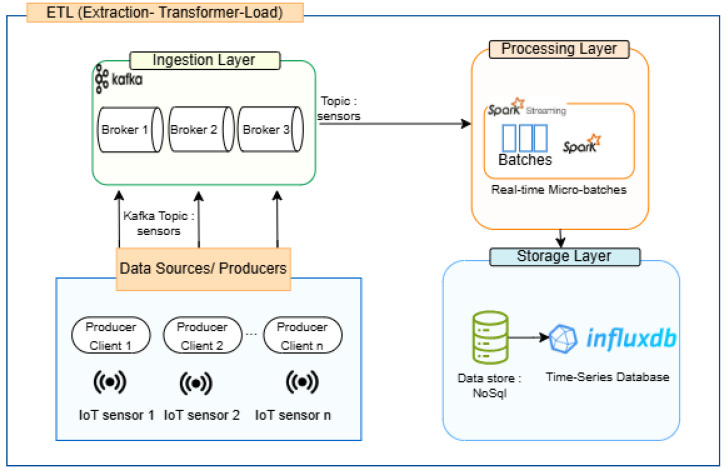
Real-time ETL pipeline architecture for IoT sensor data.

The ETL process is a fundamental method in data engineering that enables the efficient movement of data from multiple sources to a centralized system for analysis and decision-making [27]. This process is divided into three key phases, and each one plays a critical role in collecting, processing, and storing raw data in a structured format [28]. The proposed architecture is illustrated in Figure 3.

First phase: The extract phase involves retrieving data from various sources, including databases, APIs, IoT sensors, and streaming platforms like Apache Kafka. In real-time processing scenarios, this step is crucial for continuously ingesting large-scale, fast-moving data to maintain accuracy and timeliness.

Second phase: During the transform phase, the extracted data undergo cleaning, aggregation, normalization, and feature engineering to make them suitable for analysis. This step ensures data consistency by handling missing values, removing duplicates, and standardizing formats. In real-time applications, transformation is performed dynamically using frameworks like Apache Spark Structured Streaming to minimize processing delays.

Third phase: The load phase involves storing the transformed data in a target system such as a data warehouse, time-series database, or distributed storage. In real-time ETL, data are continuously streamed into these storage systems, enabling instant analytics and automated decision-making.

Taking a top-level view, we can break down the application into several modules. The main component that dictates the flow of the architecture depicted above illustrates a real-time ETL pipeline designed for ingesting, processing, and storing IoT sensor data streams in a scalable and fault-tolerant manner. The system consists of three layers: (1) the ingestion layer, where IoT data streams are ingested via Apache Kafka; (2) the processing layer, where Spark Streaming processes data in micro-batches; (3) the storage layer, where processed data are stored in a NoSQL time-series database (InfluxDB) for further analysis and model input.

The pipeline begins with Apache Kafka, a distributed event streaming platform, which acts as the ingestion layer. IoT sensors deployed across various physical environments continuously generate data, which are published to a Kafka topic (e.g., sensors). Kafka brokers (Broker 1, Broker 2, Broker 3) ensure high availability and horizontal scalability, enabling the system to handle large volumes of incoming data from multiple producers.

Next, Apache Spark Streaming connects to the Kafka topic and consumes data in micro-batches. Spark performs transformation operations such as parsing, filtering, aggregation, and feature extraction in near real-time. This stage forms the transformation component of the ETL process, making the raw sensor data ready for analysis or storage.

Once the data are processed, they are stored in InfluxDB, a high-performance, NoSQL time-series database optimized for time-stamped data. InfluxDB allows for the efficient storage, querying, and retrieval of historical IoT data, which are essential for monitoring trends, conducting analytics, and feeding downstream machine learning models. This ETL pipeline provides a robust foundation for real-time analytics and predictive maintenance in IoT systems by ensuring low-latency data flow, scalable processing, and reliable storage.

### 3.2. Machine Learning Pipeline for Real-Time Predictive Maintenance

The machine learning pipeline constitutes a critical middle layer in our end-to-end IoT predictive maintenance architecture, as shown in Figure 2. This pipeline receives preprocessed data from the ETL layer and outputs predictive analytics to the monitoring and optimization components.

Data selection focuses on capturing recent, representative operational data through rigorous filtering and sampling. EDA employs statistical profiling and visualization techniques to uncover hidden patterns and relationships in sensor data [29]. We identify key insights through correlation analysis, time-series visualization, and comprehensive statistical examination.

The feature engineering stage follows, representing a critical transformation process in predictive maintenance, where raw sensor data are converted into meaningful, predictive signals [30]. Advanced techniques extract statistical features, frequency analyses, and domain-specific indicators. Feature selection addresses high-dimensional data challenges by using Principal Component Analysis (PCA) and correlation analysis to identify the most informative variables while eliminating redundant features [31,32].

The model training stage implements diverse machine learning algorithms, including Random Forest classifiers for failure prediction, SVM for precise decision margins, XGBoost for gradient-boosted decision trees with excellent feature importance extraction, LSTM networks for remaining useful life estimation, and specialized anomaly detection models. Rigorous validation ensures reliable performance through cross-validation, confusion matrix analysis, and time-based evaluation techniques.

To maximize real-world applicability, we implement a multi-faceted approach to mitigate overfitting, as well as regularization techniques, data augmentation, and ensemble methods to enhance model generalizability. Strategies include L1 and L2 regularization, dropout layers, synthetic data generation, and advanced validation approaches that simulate real-world deployment scenarios.

**Figure 4:**
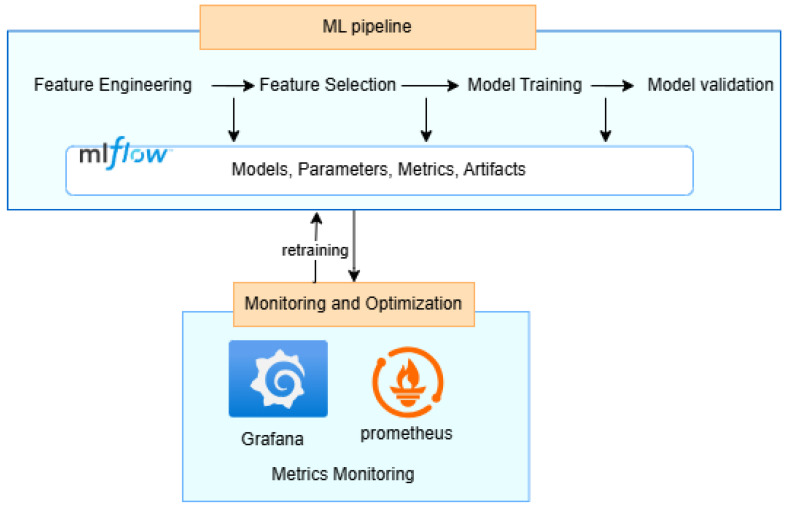
End-to-end machine learning pipeline with monitoring and retraining.

As illustrated in Figure 4, MLflow integration provides comprehensive ML lifecycle management capabilities across all stages. This integration enables experiment tracking, model versioning, and streamlined deployment processes, ensuring reproducibility and maintainability of our prediction models. The bidirectional connection between the ML pipeline and the monitoring layer enables continuous model improvement through automated retraining mechanisms. As new data accumulate and operational conditions change, the system detects performance drift and initiates retraining with the latest data, ensuring that the predictive models maintain accuracy over time and adapt to evolving equipment behavior patterns.

### 3.3. Monitoring and Visualization

The monitoring and visualization layer forms the uppermost component of our IoT analytics architecture as depicted in Figure 2. This layer provides critical capabilities for system observability, performance tracking, and user interaction with the equipment failure forecasts generated by the underlying ML pipeline.

Our implementation leverages the complementary strengths of Grafana and Prometheus to create a comprehensive monitoring ecosystem. Grafana serves as the primary visualization platform, offering intuitive dashboards that display real-time equipment status, performance metrics, and predictive maintenance alerts. These dashboards are designed with role-specific views, allowing maintenance technicians, engineers, and management to access information relevant to their responsibilities.

Prometheus functions as the underlying metrics collection and alerting system, continuously scraping performance data from all system components. This includes both infrastructure metrics (CPU utilization, memory consumption, network throughput) and application-specific metrics (model prediction accuracy, data processing latency, anomaly scores). The time-series database capability of Prometheus enables historical trend analysis and performance comparison over extended operational periods.

**Figure 5:**
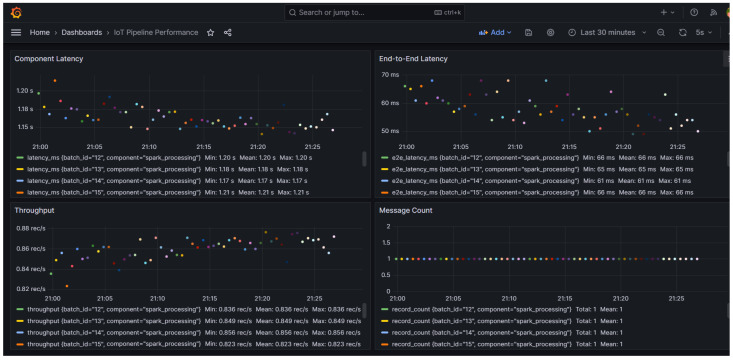
Real-time performance monitoring of IoT analytics pipeline using Grafana.

The dashboard presented in Figure 5 provides comprehensive visibility into our IoT analytics pipeline performance metrics. The Component Latency graph shows consistent Spark processing times averaging 1.15–1.18 s, with occasional fluctuations of up to 1.21 s. The end-to-end latency consistently stays between 50 and 70 ms, demonstrating efficient data flow through the Kafka messaging layer. Throughput stabilized around 0.85 records/second, showing slight improvement from earlier measurements, while the Message Count confirms that the system processes individual messages rather than batches. The substantial difference between processing latency (~1.2 s) and end-to-end latency (~60 ms) reveals that our Kafka infrastructure efficiently handles data transport, with Spark processing representing the primary performance bottleneck in the architecture.

Key Performance Indicators (KPIs) tracked by the monitoring system include prediction accuracy metrics (precision, recall, F1-score), operational metrics (mean time between failures; maintenance cost savings), and system performance metrics (data processing latency; model inference time). These KPIs provide a quantitative assessment of both the technical performance of the predictive system and its business impact.

The monitoring system implements a multi-tier alerting framework that categorizes maintenance needs based on urgency and impact. Critical alerts indicating imminent failure are propagated immediately through multiple channels (email, SMS, dashboard notifications), while less urgent maintenance recommendations are scheduled and prioritized within the maintenance workflow system. This tiered approach prevents alert fatigue while ensuring a timely response to critical situations.

Integration with the ML pipeline enables the visualization of model predictions alongside actual sensor readings, providing transparency into how the predictive algorithms make decisions, as Figure 4 describes. This transparency builds trust among maintenance personnel and facilitates the continuous improvement of both the models and the maintenance practices they inform. Moreover, by combining real-time monitoring, predictive insights, and interactive visualization, this layer transforms complex data into actionable maintenance decisions, ultimately extending equipment lifespan, reducing downtime, and optimizing maintenance resource allocation.

## 4. Real-Time System Implementation and Experimental Environment

This section outlines the implementation of the real-time system and the experimental setup used in the test scenarios.

### 4.1. Setting up Test Topic: Experimental Setting

The test environment mirrors the architecture presented in Figure 2 and Figure 3, consisting of multiple IoT sensors connected to the system for real-time data collection and analysis. Our implementation utilizes Apache Kafka for message streaming, Apache Spark for real-time processing, and InfluxDB for storing time-series data.

To establish the real-time communication infrastructure with Apache Kafka, we first initialized the Zookeeper service, which manages the Kafka cluster configuration. This was accomplished by executing the command ./bin/zookeeper-server-start.sh config/zookeeper.properties. After Zookeeper initialization, we launched the Kafka broker service using ./bin/kafka-server-start.sh config/server.properties.

We then created a dedicated Kafka topic named “data-stream” to handle our sensor data stream by running ./bin/kafka-topics.sh --create --topic data-stream --bootstrap-server localhost:9092 --partitions 1 --replication-factor 1. This topic serves as the communication channel between our data collection components and the processing pipeline.

For our data collection pipeline, we activated a Python virtual environment with the command source myenv/bin/activate and executed our data collection script python Data_collector_kafka.py. This script implements both the data collection logic and the Kafka Producer functionality, sending sensor readings to the “Sensor_data” topic for consumption by downstream processing components.

To process the streaming data, we deployed a Spark application with the necessary Kafka connector package using the command spark-submit --packages org.apache.spark:spark-sql-kafka-0-10_2.12:3.2.4 kafka_consumer_spark.py. This Spark job continuously consumes messages from the Kafka topic, processes them in real-time, and prepares them for storage and analysis.

For data persistence and time-series analytics, we utilized InfluxDB. The InfluxDB interface was accessed through an SSH tunnel to ensure secure communication by executing ssh -L 8086:localhost:8086 user@hpc-login.marwan.ma. This command established port forwarding from our local machine to the InfluxDB server running on port 8086, enabling access to the InfluxDB web interface at http://localhost:8086/orgs/e2ca41fdf26b163e/load-data/buckets.

All experiments were conducted on MARWAN’s high-performance computing (HPC) infrastructure [33], which provided the necessary computational resources for real-time data processing and predictive modeling. The HPC environment ensured consistent performance across multiple test scenarios and supported the scalability requirements of our IoT analytics pipeline.

### 4.2. Real-Time Communication with Apache Kafka and Spark

The integration of Apache Kafka and Apache Spark Structured Streaming is a key component in enabling real-time data processing for IoT-based systems. Kafka acts as a distributed message broker, efficiently handling high-throughput data streams from multiple IoT sensors. It ensures fault tolerance, scalability, and durability by organizing incoming sensor data into topics, which can be consumed by downstream processing engines.

**Figure 6:**
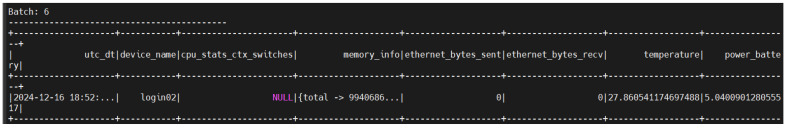
Batches of real-time data using Spark.

On the other hand, Spark Structured Streaming serves as the real-time processing layer, consuming data from Kafka topics and applying transformations such as cleaning, aggregation, and feature extraction. Spark processes data as micro-batches as shown in Figure 6, where streaming data are divided into small, fixed intervals, ensuring efficient processing while maintaining near real-time performance. This allows for continuous and near-instantaneous data processing, ensuring that only refined, structured, and relevant information is forwarded to the storage layer. In this methodology, the processed data are stored in InfluxDB, a time-series database optimized for handling high-frequency IoT data, enabling efficient querying and analytics.

### 4.3. NoSql Database Influxdb

Efficiently managing IoT data requires selecting the right database system, as IoT environments generate large-scale, high-frequency data streams. Choosing the optimal database for IoT applications can be challenging due to varying system requirements. Several critical factors must be considered, including scalability, performance, flexible schema design, analytical capabilities, security, and cost-effectiveness. Unlike traditional relational databases, NoSQL databases offer greater flexibility and scalability, making them well suited for handling time-series data from IoT sensors [34].

Among various NoSQL solutions, InfluxDB, Cassandra, MongoDB, and Redis are widely used for managing time-series data. In this study, InfluxDB is adopted as a fundamental component in the ETL process, specifically in the load phase, to efficiently store and manage IoT sensor data. InfluxDB is an open-source time-series database (TSDB) designed for high-performance storage, retrieval, and analysis of time-stamped data. Its schema-less architecture enables the efficient handling of continuous data streams, making it ideal for real-time analytics, predictive maintenance, and IoT monitoring systems.

One of the key strengths of InfluxDB is its ability to physically order data by time, ensuring the efficient retrieval and processing of historical trends. It also supports retention policies, allowing automatic data aggregation or deletion based on predefined time frames, which helps manage storage efficiency. Additionally, InfluxDB provides continuous queries, enabling real-time computations on incoming data streams rather than relying solely on batch processing. It follows a hybrid data model, where values are stored in a row-based format, while tags (indexes) are managed in a columnar fashion, optimizing both storage and query performance. These features make InfluxDB a powerful choice for IoT-based predictive maintenance and real-time data analytics [35].

**Figure 7:**
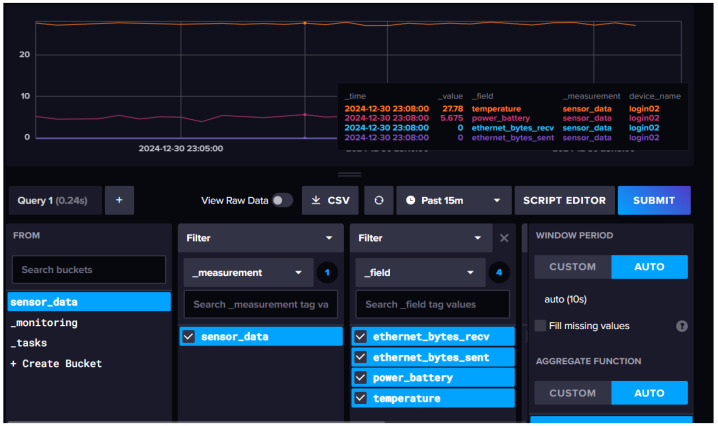
Influxdb interface presents features for streaming data.

Additionally, InfluxDB provides a web-based UI for querying, visualizing, and managing time-series data. It supports InfluxQL, an SQL-like query language with built-in time-based functions, and integrates with tools like Chronograf for real-time monitoring and Telegraf for seamless IoT data ingestion [36]. Figure 7 illustrates a UI visualization of our time-series dataset project.

## 5. Conclusions

This research paper proposed an end-to-end architecture for real-time IoT analytics and predictive maintenance, integrating Apache Kafka, Apache Spark Structured Streaming, and InfluxDB. Our experimental results confirm the practicality of combining these technologies to build a comprehensive predictive maintenance solution applicable across diverse industrial domains. The implementation successfully demonstrates the architecture’s capability to process streaming sensor data, apply machine learning for failure prediction, and transform complex recommendations through monitoring and visualization, establishing the effectiveness of stream processing and ML pipelines in IoT analytics systems requiring real-time responses to equipment condition changes.

Recognizing the current architectural limitations, future research will focus on three key areas of improvement: optimizing the machine learning pipeline through advanced feature engineering and model selection strategies; implementing Self-Supervised Learning techniques to leverage unlabeled sensor data; enhancing system resilience through automated performance optimization and robust security protocols to protect against potential adversarial attacks in industrial IoT environments.

These improvements represent more than technical refinements; they signify a critical step toward realizing the full potential of intelligent, predictive IoT systems that can dynamically adapt, self-optimize, and provide unprecedented insights across complex industrial landscapes.

## Data Availability

Data are contained within the article.

## Abbreviations

The following abbreviations are used in this manuscript:

IoT	Internet of Things
ETL	Extract Transform Load
API	Application Programming Interface
PDM	Predictive Maintenance
ML	Machine learning
AoI	Age of Information
SVM	Support Vector Machine
LSTM	Long Short-Term Memory
CNN	Convolutional Neural Network
Bi-LSTM	Bidirectional Long Short-Term Memory
SSL	Self-Supervised Learning
CV	Computer Vision
NLP	Natural Language Processing
GBMs	Gradient Boosting Machines
XGBoost	eXtreme Gradient Boosting
EDA	Exploratory Data Analysis
NoSQL	Not Only Structured Query Language
PCA	Principal Component Analysis
TSDB	Time-Series Database
SMS	Short Message Service
KPIs	Key Performance Indicators
SSH	Secure Shell
